# A high‐resolution global flood hazard model†

**DOI:** 10.1002/2015WR016954

**Published:** 2015-09-12

**Authors:** Christopher C. Sampson, Andrew M. Smith, Paul D. Bates, Jeffrey C. Neal, Lorenzo Alfieri, Jim E. Freer

**Affiliations:** ^1^ School of Geographical Sciences, University of BristolBristolUK; ^2^ European Commission, Joint Research CentreIspraItaly

**Keywords:** flooding, large‐scale modeling, global, hydraulic

## Abstract

Floods are a natural hazard that affect communities worldwide, but to date the vast majority of flood hazard research and mapping has been undertaken by wealthy developed nations. As populations and economies have grown across the developing world, so too has demand from governments, businesses, and NGOs for modeled flood hazard data in these data‐scarce regions. We identify six key challenges faced when developing a flood hazard model that can be applied globally and present a framework methodology that leverages recent cross‐disciplinary advances to tackle each challenge. The model produces return period flood hazard maps at ∼90 m resolution for the whole terrestrial land surface between 56°S and 60°N, and results are validated against high‐resolution government flood hazard data sets from the UK and Canada. The global model is shown to capture between two thirds and three quarters of the area determined to be at risk in the benchmark data without generating excessive false positive predictions. When aggregated to ∼1 km, mean absolute error in flooded fraction falls to ∼5%. The full complexity global model contains an automatically parameterized subgrid channel network, and comparison to both a simplified 2‐D only variant and an independently developed pan‐European model shows the explicit inclusion of channels to be a critical contributor to improved model performance. While careful processing of existing global terrain data sets enables reasonable model performance in urban areas, adoption of forthcoming next‐generation global terrain data sets will offer the best prospect for a step‐change improvement in model performance.

## Key Points

- High‐resolution flood hazard model that employs globally available data sets
- Quantitative assessment of model performance relative to benchmark local data
- Performance adequate for certain real world applications in data‐scarce regions

## 1 Introduction

The repeated occurrence of high‐profile flood events (e.g., Australia and Thailand in 2011; central Europe in 2013; and India and Pakistan in 2014) has resulted in sustained public, commercial, political, and scientific interest in flooding. Historically, our understanding of global flood hazard has been limited by the poor monitoring of river networks in many regions [*Fekete et al*., 2002], the restricted spatiotemporal coverage of suitable remotely sensed data sets used to map flood extent [*Bates et al*., 2014; *Prigent et al*., 2007] and the limited resolution and process representation of regional to global scale computer flood models [*Bell et al*., 2007; *Ngo‐Duc et al*., 2007]. Looking to the future, the continued expansion of cities located on river floodplains and coastal deltas due to population growth and migration is expected to produce a significant increase in flood risk over the coming decades [*Hirabayashi et al*., 2013; *Jongman et al*., 2012]. Furthermore, scenario‐based simulations using climate models do indicate the possibility of increasing future flood risk in many regions due to climate change, although there is considerable variability between models and scenarios [*Arnell and Gosling*, 2014]. This prospect is of particular concern to international humanitarian and development organizations such as the World Bank and the UN World Food Programme, as well as to the global (re)insurance market. Recent losses have proven significant: household losses resulting from the summer 2007 floods in the UK reached £2.5 billion, with business losses accounting for a further £1 billion [*Chatterton et al*., 2010]. Estimates of the total economic losses from the Australian and Thailand events of 2011 range between USD 2.8–6.1 billion and USD 30–40 billion, respectively [*Munich Re*, 2012; *Swiss Re*, 2012].

Flood hazard maps are designed to indicate the probability of flooding over space and serve as a critical decision‐making tool for a range of end users including building/infrastructure developers and disaster response planners. However, such maps do not exist for much of the developing world due to the extremely high data and computational requirements of the engineering hydraulic models that have traditionally been used in their production. Existing research toward more tractable simplified global scale models of surface water flows has typically been limited to hydrological routing schemes that are driven by regional or global climate models. One of the more sophisticated schemes of this type involved linking the Total Runoff Integrating Pathways 2 (TRIP2) global river network [*Ngo‐Duc et al*., 2007] to the European Centre for Medium Range Weather Forecasts (ECMWF) land surface model (HTESSEL) in order to evaluate the feasibility of modeling global river runoff at a daily time scale [*Pappenberger et al*., 2010]. The study concluded that although the HTESSEL/TRIP2 model is of use in the verification of the ECMWF land surface scheme, such a model could only provide indicative advice pertaining to extreme discharges and flood warnings even when likely future improvements are taken into account. *Yamazaki et al*. [2011] presented the global river routing model CaMa‐Flood in which the relationship linking water storage, water level, and flooded area is based on subgrid‐scale topographic parameters derived from a high‐resolution digital elevation model (DEM). The model uses 1° resolution runoff data, generated by a land surface model driven by reanalysis and observed data, to calculate flow between grid points along the river network using a local inertia shallow water equation. Each grid point has a channel and floodplain reservoir associated with it, with the floodplain reservoir consisting of the river unit catchment for that point. Water level and flooded fraction for each grid point are calculated from storage volume using relationships derived from the topographic parameters. The model showed good correlation with satellite observations at the daily scale and demonstrated improved performance relative to a kinematic realization of the model over most of the 30 river basins tested. However, as the driving land surface model is poor at characterizing extremes, and the routing model provides a coarse resolution (∼15 arc min or ∼25 km) estimate of flooded fraction rather than a high‐resolution map of water depths, its applicability within a flood hazard context is limited. GLOFRIS, a recent framework for global river flood risk assessment [*Winsemius et al*., 2013] that uses climate forcing data sets (precipitation, temperature, and potential evaporation) to drive a global hydrological model (PCR‐GLOBWB) and kinematic routing model (PCR‐GLOBWB dynamic routing), was coupled to an inundation downscaling routine to produce estimates of flood extent and depth. The mass‐conservative downscaling component allows the water volume in each 0.5° routing cell to be converted into high‐resolution (∼1 km) water depths using an algorithm that identifies river cells within the 1 km resolution DEM and iteratively increases the water level within each of these cells, allowing water to spread into connected upstream floodplain cells, until the correct volume is reached. The downscaler is therefore conceptually similar to other volume spreading algorithms used at reach scales [e.g., *Gouldby et al*., 2008], and while highly expedient, the skill of such schemes under conditions of relatively poor quality topography is uncertain given the demonstrated importance of process representation in other 2‐D hydraulic models [*Neal et al*., 2012; *Sampson et al*., 2012]. Although simulations of exposed population and exposed urban assets for the period 1990–2000 showed fair correlation with observed fatalities and economic losses when aggregated to the global scale [*Ward et al*., 2013], the local skill of the method was unclear in the presented qualitative validation over Bangladesh [*Winsemius et al*., 2013] and therefore its ability to characterize flood hazard at high resolution remains to be demonstrated and quantified.

Over recent years, the large gap that exists between simplified large‐scale approaches and detailed reach scale hydraulic models has begun to close as the result of significant research advances and increasing computational and data resources. A number of detailed hydraulic models (e.g., spatial resolutions of 250–1000 m) have been constructed for large river reaches in data sparse regions including the Amazon [*da Paz et al*., 2011; *de Paiva et al*., 2013], the Ob [*Biancamaria et al*., 2009], the Niger [*Neal et al*., 2012], the Congo [*Jung et al*., 2010], and the Zambezi [*Schumann et al*., 2013]. These approaches vary in complexity from coupled 1‐D/floodplain storage models [*de Paiva et al*., 2013], through coupled 1‐D channel/simplified 2‐D floodplain models [*Biancamaria et al*., 2009; *da Paz et al*., 2011], to 2‐D models with subgrid representations of minor channels [*Neal et al*., 2012; *Schumann et al*., 2013]. However, continental‐scale studies that employ detailed hydraulic models are scarce due to the difficulties of data availability and computational expense. *Feyen et al*. [2012] produced estimates of fluvial flood risk in Europe at local scales (∼100 m) using a model cascade that converted estimated river water depths into inundation extents using a simple planar approximation for inundation extent. A more recent study produced by the European Commission's Joint Research Centre (hereon referred to as the EC‐JRC model) used a combination of distributed hydrological and hydraulic models, driven using observed meteorological data, to derive a 100 year return period pan‐European flood map in which inundation extents were simulated using over 37,000 two‐dimensional hydraulic models, each representing a ∼5km reach of main river channel [*Alfieri et al*., 2013]. These studies were feasible over Europe due to the relative abundance of data that can be used to characterize its catchments; no such studies yet exist for developing regions where the need for flood hazard maps is greatest.

In this paper, we propose a methodology that enables the construction of a global flood model for flood hazard assessment at ∼90 m spatial resolution where local detailed data and observations are not available. Models of this type are already being developed by the private sector in the insurance market for underwriting purposes in developing regions, but the detailed methods underlying such commercial models are not always clear and public domain validation exercises are limited. Validation of these methods is crucial to ensure that they can be applied appropriately through a proper understanding of their strengths and limitations. We argue there are six key challenges that need to be overcome to allow the development of such models, and that the state of science has advanced to the point whereby each of these challenges can now be tackled. The resulting global model is sufficiently resolved to enable direct benchmarking against high‐resolution local and national flood hazard data sets—to our knowledge the first time such an exercise has been undertaken for a model of this type. The framework and methods presented here should be considered a starting point, and we identify a number of areas where significant improvement may be achieved through targeted future research.

## 2. Global Flood Hazard Modeling: Six Key Challenges

**Figure 1:**
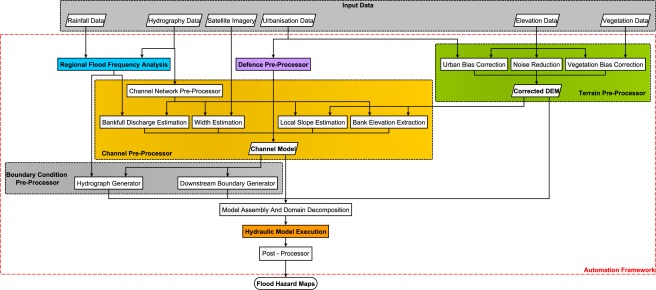
Conceptual flowchart of global flood hazard model framework. The colors associate areas of the flowchart to the six key challenges presented in this paper: global terrain data (green); extreme flow generation (blue); global river network and geometry (yellow); flood defenses (purple); computational hydraulic engine (orange); and automation framework (red).

Below we have identified six key challenges that need to be solved to enable a global flood model to be built. The bracketed colors in the list below associate each challenge with a region of the methodological flowchart (Figure 1).

1. Global terrain data (green).
2. Extreme flow generation (blue).
3. Global river network and geometry (yellow).
4. Flood defenses (purple).
5. Computational hydraulic engine (orange).
6. Automation framework (red).

For each challenge, we review relevant recent advances in the literature before presenting the approach we have adopted. It should be noted at this point that the above is not an exhaustive list of challenges for this topic, and others not yet handled by this framework are introduced as limitations in the discussion section.

### 1 Global Terrain Data

#### 1.1 Review

Currently, the Shuttle Radar Topography Mission (SRTM) [*Farr et al*., 2007; *Rabus et al*., 2003] is considered to be the best openly available digital elevation data set that offers near‐global coverage [*Hirt et al*., 2010; *Jing et al*., 2013; *Rexer and Hirt*, 2014]. The primary alternative to SRTM is ASTER [*Abrams*, 2000; *Fujisada et al*., 2012], but despite the high nominal resolution of ASTER (30 m), SRTM is considered superior for flood modeling as it contains fewer surface artifacts and generally exhibits a lower vertical RMSE against ground truth data [*Jing et al*., 2013; *Rexer and Hirt*, 2014]. The SRTM digital elevation model is available between 56°S to 60°N at a resolution of 3 arc sec (∼90 m), and until recently the native 1 arc sec (∼30 m) data have only been available for the United States and Australia (the latter of which is only available for public use in bare‐earth form). However, recent military declassification of the data means that full global release of SRTM at 1 arc sec is now scheduled for 2015, although it will be necessary to evaluate the data noise properties at 1 arc sec before applying this data to flood hazard models. However, a number of problems exist with SRTM data that impede its applicability to flood modeling, including voids, speckle, and large biases in forested and urban areas. Voids are typically the least problematic for flood modeling applications as they typically occur in areas of very steep relief where topographic shadowing occurs, over permanent water bodies due to the specular reflection of water, or in desert regions due to complexities in the dielectric constant [*Kervyn*, 2001; *Reuter et al*., 2007]. A number of void‐filling interpolation methods exist that are applicable to SRTM (for a review see *Reuter et al*. [2007]), and void‐filled SRTM data sets are now freely available, including the Hydrosheds data set [*Lehner et al*., 2008], the Consortium for Spatial Information (CGIAR‐CSI) Version 4 release [*Jarvis et al*., 2008], and the Land Processes Distributed Active Archive Centre (LP DAAC) SRTM Version 3.0 [*Kobrick*, 2013]. Speckle, or noise, is a problem that afflicts synthetic aperture radar (SAR) products such as SRTM due to instrument thermal noise, and a number of methods have been applied to SRTM that aim to reduce noise while preserving genuine features in the terrain [*Gallant*, 2011; *Gallant and Read*, 2009; *Hutchinson et al*., 2009; *Stevenson et al*., 2010]. Alternatively, as noise in SRTM is dominated by short correlation lengths, coarsening the grid scale yields a linear reduction in noise that is proportional to 1/√*n*, where *n* is the number of cells being averaged [*Rodriguez et al*., 2006; *Wilson et al*., 2007]. Despite these problems, remarkable value in SRTM data has been demonstrated for use with flood modeling. *Sanders* [2007] showed that even on the fairly narrow (∼200 m wide) Santa Clara River in the U.S., simulations undertaken using SRTM yielded flood zones that were within 25% of those simulated using a 1/9 arc sec LiDAR DEM.

One further problem that has received significant attention in recent years is the difficulty of obtaining a bare‐earth DEM from SRTM in vegetated areas. The inability of SAR to fully penetrate the tree canopy results in significant positive biases in the SRTM ground elevation over densely forested regions [*Brown et al*., 2010], and a similar problem occurs in urban areas where radar returns from the roofs of buildings are more common than returns from ground level. Recently released global estimates of forest canopy heights [*Lefsky*, 2010; *Simard et al*., 2011] enable systematic corrections to be made to SRTM data, yielding significant improvements to hydraulic models run in tropical forested catchments [*Baugh et al*., 2013].

#### 1.2 Method

**Figure 2:**
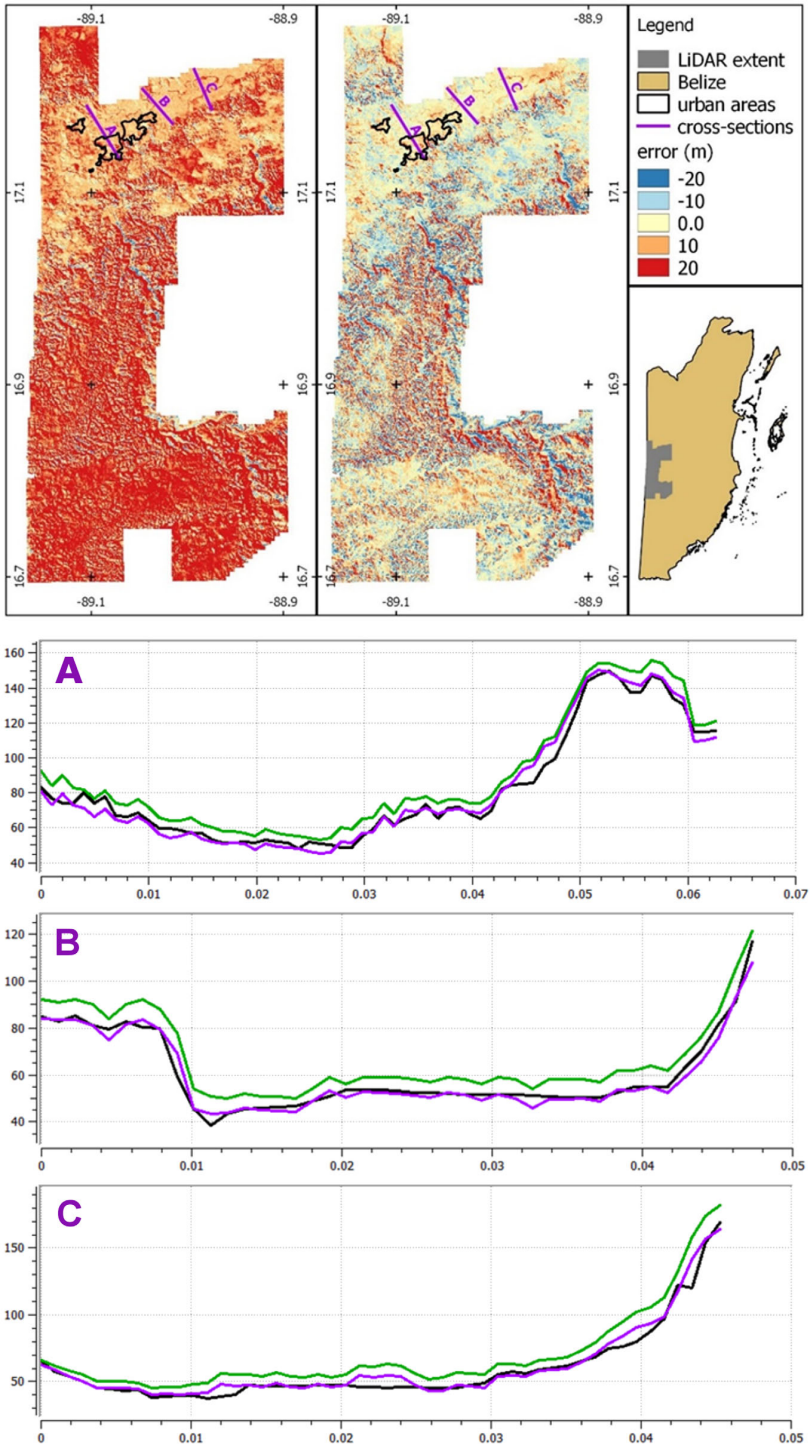
Comparison of (top left) raw SRTM DEM to LiDAR and (top middle) corrected SRTM to LiDAR for Western Belize. Cross sections (a, b, and c) transect the Belize River valley and compare the LiDAR elevation profile (black) to the uncorrected SRTM profile (green) and corrected SRTM profile (purple).

The void‐filled Hydrosheds variant of SRTM is used as the base data set for this model [*Lehner et al*., 2008], with both 3 and 30 arc sec versions being used (see below). Several corrections are then applied to the 3 arc sec data. In areas where vegetation bias is a significant issue (particularly tropical forests), a systematic vegetation correction is applied [*Baugh et al*., 2013; *Simard et al*., 2011]. A similar systematic correction is used in urban areas, where a measure of urbanization [e.g., *Elvidge et al*., 2007] is used to control a moving window filter. This approach is premised on the assumption that as urbanization increases, the proportion of pixels in a local sample that represent ground level decreases. The filter constructs a distribution of elevations from pixels within the window, assigning a value to the central pixel based on a quantile of the distribution selected according to the degree of urbanization; the filter was calibrated by comparing bare‐earth LIDAR elevations to SRTM in a number of urban areas. Noise reduction is then performed using a feature preserving adaptive smoothing algorithm [*Gallant*, 2011]. In order to ensure consistency across the 3 and 30 arc sec data sets, each 30 arc sec cell is corrected by the mean of corrections across the 100 3 arc sec cells within it. The result is a locally smoothed DEM that exhibits reduced noise relative to raw SRTM while maintaining real topographic features whose vertical scale exceeds the noise threshold. An exemplar validation of the method is presented in Figure 2 for an area of Belize for which LiDAR data were available [*Chase et al*., 2014]. The predominant land‐cover across most of the test area is tropical rainforest; the river floodplain across which the cross sections are defined has been cleared of vegetation and is a mix of agricultural and urban land uses. Accompanying statistics of elevation RMSE and bias are shown in Table 1. The results show that the method significantly reduces RMSE and bias, although it is not able remove all the errors that SRTM suffers from in steep sloped areas, as seen in the residual red and blue in the mountainous areas (opposite sides of the valleys) in the vegetation corrected SRTM comparison in Figure 2b. However, these residuals are relatively insignificant for flood hazard modeling as most floodplains are not in steeply sloping areas, and the large reduction in overall bias from 15.8 to −0.1 m demonstrates the critical necessity of such correction for flood hazard modeling.

**Table 1:** Validation Statistics for Comparison of SRTM With LiDAR Data

Comparison Statistics (m)	All Elevations	Elevations <100 m	Urban Areas
Raw DEM RMSE	20.64	12.22	10.09
Corrected DEM RMSE	12.64	6.87	6.05
RAW DEM bias	15.80	9.87	8.58
Corrected DEM bias	−0.10	1.16	3.12

### 2 Extreme Flow Generation

#### 2.1 Review

There are two main approaches to estimating extreme flood generating river discharge, namely from continuous simulation using hydrological models and flood frequency analysis. Hydrological models transform an input precipitation time series into an output river discharge time series and range in complexity from simple spatially lumped models such as HBV [*Bergstrom and Forsman*, 1973] to complex, spatially distributed models such as the Systeme Hydrologique Europeen [*Abbott et al*., 1986a, 1986bb]. However, two fundamental problems exist with applying hydrological models at regional to global scales for flood hazard analysis. The first is the issue of precipitation data quality. A number of global precipitation data sets do exist, based on satellite data [e.g., *Huffman et al*., 2007; *Joyce et al*., 2004], model reanalysis [e.g., *Dee et al*., 2011] or gauge records [e.g., *Xie et al*., 2007]. However, these products are known to have limitations that are of particular concern to flood modeling including spatially variable biases [*Kidd et al*., 2012], poor correlation with ground gauges at short (∼daily) time scales [*Chen et al*., 2014; *Cohen Liechti et al*., 2012], poor representation of spatial variability over smaller catchments [*He et al*., 2009] and a tendency to underestimate heavy rainfall [*Chen et al*., 2014; *Gao and Liu*, 2013]. *Sampson et al*. [2014] evaluated the effect of these differences on flood risk using a cascade model structure that replicates an insurance catastrophe model and found estimates of monetary loss from flooding to vary by more than an order of magnitude depending on whether the cascade was driven with gauge, radar, satellite, or reanalysis data. The second problem relates to the use of hydrological models where calibration data do not exist, as it is well known that the prediction of extreme flows in ungauged catchments remains a key challenge to the field of hydrology [*Blöschl et al*., 2013]. Although databases such as that curated by the Global Runoff Data Centre (GRDC) have started to collate international flow records that could be used for calibration, data are concentrated on large rivers in North America, western Europe, and Japan, and the limited records that are available from developing regions are unevenly distributed and typically short. As much of the value of a global flood model would be in regions where data are scarce, the combination of highly uncertain precipitation data coupled to uncalibrated hydrological models would be acute.

The alternative approach is to develop a regionalized flood frequency analysis (RFFA). Although differing in their specific methods, regional flood frequency analyses work on the assumption that data from data‐rich regions can be transferred to data poor ones. More specifically, these methods assume that catchments with similar characteristics will also exhibit similar flood frequency statistics. RFFA are well known in the literature and have been widely used to provide estimates of discharge in data poor regions [*Gaume et al*., 2010; *Merz and Blöschl*, 2009; *Zaman et al*., 2012]. However, RFFA studies have largely been limited to specific regions [*Farquharson et al*., 1992; *Meigh et al*., 1997; *Merz and Blöschl*, 2009] as their application over larger scales has been precluded by the absence of globally consistent observational data sets. Although the advent of such data sets has begun to change this landscape, these data are still restricted to basic catchment descriptors that only allow for simplistic methods to be undertaken [*Smith et al*., 2015]. The application of RFFA is also limited by uncertainties in observed discharge records which are known to be significant for extreme flows [*Pappenberger et al*., 2006]. Moreover, the estimation of extreme flow behavior using catchment characteristics is further hindered by human modification of river systems that change natural extreme flow behavior. An additional facet of RFFA is that such methods can also been employed in conjunction with continuous simulation using hydrological models to characterize flow at higher‐order return periods [*Cameron et al*., 1999].

#### 2.2 Method

As previously indicated, globally consistent observational data sets that allow simplistic catchment characteristics, such as area and rainfall, to be defined are now emerging. Although, the GRDC database has limited coverage in some regions, it contains sufficient quantity of data from all major climatic zones. Combined, these data sets enable RFFA to be applied at the global scale and subsequently allow preliminary discharge estimation with global coverage.

The method used here follows that described by *Smith et al*. [2015] using a hybrid‐clustering approach in conjunction with a flood‐index methodology. The method uses catchment descriptors of climate class, upstream annual rainfall, and catchment area and proceeds as follows: data from available stations are subdivided into the five main categories of the Koppen‐Geiger climate classification [*Kottek et al*., 2006]. A clustering method is then used to pool together suitably homogenous catchments; the clustering method used for regionalization is a combination of Ward's algorithm and *k*‐means clustering [*Ramachandra Rao and Srinivas*, 2006]. A flood estimation index approach is then applied, providing estimates of the mean annual flood (MAF) and extreme value distributions or flood frequency curves for each of the pooled regions. When combined with the index flood, MAF, the flood frequency curves or growth curves provide a basic means of flood estimation for any region [*Meigh et al*., 1997; *Zaman et al*., 2012]. These methods are applied to a global data set of over 3000 gauging stations, sourced from the Global Runoff Data Centre (GRDC) and from the United States Geologic Survey (USGS) stream gauge network (http://nwis.waterdata.usgs.gov/nwis). Although the ability of this approach to provide detailed, localized discharge estimates is limited by the simplicity of the methods, significant uncertainties in the discharge record and the complexity of anthropogenically modified river systems, these methods have demonstrated skill in providing first‐order discharge estimates in data poor regions [*Padi et al*., 2011; *Smith et al*., 2015]. Estimating extreme discharge via this method is subject to significant uncertainty, as is the case with all generalized global methods; although global mean errors of ∼80% were reported by *Smith et al*. [2015], far larger errors were also reported, in some cases >300%. Because such errors are currently unavoidable in global scale models, discharge estimation bias is explicitly accounted for in the modeling framework by scaling channel conveyance within the hydraulic model according to the estimated MAF. These procedures are described in the following section.

The above methods are based upon annual maxima (AMAX) data, and therefore enable AMAX discharge with an associated recurrence interval to be estimated. However, they do not constitute flood hydrographs that are required in order to enable hydrodynamic simulations to be undertaken. A simple design hydrograph is therefore generated using the rational hydrograph method, where the time to concentration for each catchment is calculated using Manning's equation to estimate routing velocity along the river network.

Although the RFFA provides estimates of return period discharge for rivers and streams, it does not allow for accurate discharge estimation in small channels in which flooding is generally driven by intense local precipitation. For small channels (catchment area <50 km^2^), an alternative method is required, with flow generated by raining directly on to the DEM (“rain‐on‐grid”). Therefore, in addition to the estimation of discharge, methods were also required for the estimation of extreme rainfall. This was achieved via a similar approach to the RFFA, only Intensity‐Duration‐Frequency (IDF) relationships were used as opposed to discharge data. IDF relationships are a standard engineering‐based method of estimating the intensity, duration, and frequency of extreme rainfall events. In total, IDF data from ∼200 locations were used. The procedure is as follows: IDF data from various locations around the world were pooled together and again partitioned into the various categories of the Koppen‐Geiger climate classifications [*Kottek et al*., 2006]. Within each climate classification, regressions between annual average rainfall (AAR) and the IDF data were estimated, with the AAR acting as an additional climate descriptor, estimating extreme rainfall using the most appropriate IDF relationships within each climate classification.

Although preliminary validation confirmed correlation between AAR and extreme rainfall at locations across the planet where data were available, this link has not been subjected to a detailed published study. Therefore, the estimates of extreme rainfall derived using this method should be considered first order, and may be subject to significant errors. We do not suggest that this method is able to provide robust estimates of return period rainfall at all locations globally as local‐scale features will inevitably have a significant influence. However, when considering whether this method is fit for purpose, it is important to consider these errors relative to other uncertainties. Of particular relevance here is that the “rain‐on‐grid” simulations are undertaken directly on a 3 arc sec resolution SRTM DEM, and at this resolution, the SRTM noise error (which is of the order of meters even on flat river floodplains) is likely to dominate boundary condition uncertainties (such as precipitation intensity) within the hydraulic model. Under such conditions, more accurate extreme rainfall estimates may not yield improvements in simulated flood hazard due to the DEM noise, and we therefore consider the method appropriate for this application.

### 3 Global River Network and Geometry

#### 3.1 Review

As described by *Neal et al*. [2012], the inclusion of a channel network within large‐scale flood models is necessary to achieve acceptable simulations of flood depth, extent, and dynamics. The Hydrosheds project [*Lehner et al*., 2008] used the SRTM DEM to derive a global hydrography data set which includes hydrologically conditioned DEMs, catchment delineations, river networks, flow direction arrays, and accumulating area arrays. The underlying SRTM DEM was subjected to extensive hydrological conditioning and manual editing to ensure channel connectivity through narrow valleys and other complex topographies, and the result is a near‐global river database available at 3 and 30 arc sec resolutions. The data set has been used in a range of studies including global river routing [*Gong et al*., 2011], operational flood forecasting [*Li et al*., 2009], and global river width analysis [*Yamazaki et al*., 2014] and typically provides a baseline data set for locating river channels within large‐scale hydraulic models.

Recently, a number of studies have capitalized on the availability of remotely sensed data to generate regional to global estimates of river widths, either by building on the classical geomorphological relationships of *Leopold and Maddock* [1953] at large scales [*Andreadis et al*., 2013] or through satellite image processing techniques [*Gleason and Smith*, 2014; *Miller et al*., 2014; *O'Loughlin et al*., 2013; *Pavelsky and Smith*, 2008; *Yamazaki et al*., 2014]. These methods can be augmented by coupling river network data to web‐based imagery services such as Google maps or Bing maps, allowing rapid manual surveys of river widths using browser‐based distance measurement tools. This allows more accurate river width profiles to be created for areas of particular interest or complexity such as major urban centers or coastal deltas.

#### 3.2 Method

In order to simulate extreme flows in river channels, it is necessary to estimate the conveyance capacity of the river, characterized by the river width and depth, as an accurate representation of channel bankfull capacity is necessary for behavioral simulations of flood events [*Fewtrell et al*., 2011]. River widths are estimated using a hybrid geomorphological/web‐survey technique in which river widths along major rivers within a domain are measured and recorded along with their corresponding upstream accumulating areas. A spline is then fitted through the data that allows the width of a cell to be estimated based on its upstream area; specific splines are produced for major rivers, and the data are pooled to create a generalized spline for use along unsurveyed rivers.

River depth is the most difficult parameter to estimate as it is not yet possible to measure this remotely on large scales. Where detailed local data are not available, it is necessary to infer an appropriate estimate of river depth at any location on the river network from available data. By assuming a bankfull discharge return period of approximately 1 in 2 years [*Andreadis et al*., 2013; *Harman et al*., 2008; *Leopold*, 1994], the flow generation procedure described in the preceding section is able to yield an estimate of bankfull discharge. By combining bankfull discharge, channel width, and an estimate of slope calculated from the DEM, it is then possible to produce an estimate of channel depth using Manning's equation. Linking channel geometry to discharge return period in this manner ensures that the channels are appropriately sized for the flows being simulated, mitigating against the problem of gross mismatches between discharge and channel conveyance.

The final stage is to decompose the model domain into individual reaches for simulation. Reaches are not arbitrary in length but are generated automatically according to a threshold difference in the modeled mean annual flood (MAF) between the upstream and downstream boundaries of the reach. Starting at the downstream end, the model tracks up the river network until the point is reached at which the MAF falls a threshold percentage below the downstream MAF (this threshold is an adjustable parameter; 5% is used here). The RFFA is then used to generate a hydrograph for the downstream cell of the reach; the hydrograph is distributed among the upstream cell and any incoming tributaries according to their respective upstream catchment areas to ensure that the volume flowing into the reach at any point matches that of the RFFA‐generated downstream boundary. Each reach is simulated independently to prevent autocorrelation issues in the lower reaches of catchments.

### 4 Flood Defenses

#### 4.1 Review

If the global flood hazard model is to be used for flood risk estimation where more detailed resources are not available, then it is necessary to consider the impact of flood defenses as the majority of risk will be situated in urban areas where the potential for damage (“exposure”) and defenses (“mitigation”) is highest. Representing flood defenses in a global flood model is challenging because the engineered features themselves will typically be far smaller than the model grid scale and little or no data will be available to characterize them in most locations; the latter limitation also applies to the operating rules of reservoirs that are often designed to mediate fluvial flood risk. However, it is a fair assumption that most urban areas situated on floodplains will have some degree of flood defense, and that the level of defense will be related to the wealth and development of the country in which it is located. Literature relating to global flood defenses is scarce; a recent World Bank led study attempted to characterize defense levels in major coastal cities [*Hallegatte et al*., 2013] but literature relating to fluvial flood defenses normally relates to individual sites or catchments [e.g., *Brandimarte and Di Baldassarre*, 2012; *te Linde et al*., 2011; *Wesselink et al*., 2013]. One available option is to combine literature review, experience, and local knowledge (where available) to develop a database of defense levels for known locations. These can then be regressed against socioeconomic variables such as GDP [*Feyen et al*., 2012] or population density to provide a means of estimating defense standards elsewhere. The spatial distribution of defense standards can be estimated in a similar way by regressing known defense locations against measures of urbanization such as the luminosity‐based impervious surface area (ISA) data set [*Elvidge et al*., 2007]. A similar method to this has recently been applied in the EU at finer scales using catchment level descriptors based on potential losses from an insurance database [*Jongman et al*., 2014], but at global scale the data do not yet exist to replicate the Jongman et al.'s approach.

#### 4.2 Method

The benchmark data used in this study assume that defenses have failed, and so a defense method is not implemented here. However, it is possible to incorporate defenses within the framework by increasing channel conveyance according to an estimate of local defense standards. A method for estimating these standards based on socioeconomic factors and remotely sensed urbanization data sets will receive full treatment in a future study.

### 5 Computational Hydraulic Engine

#### 5.1 Review

The emergence of highly efficient algorithms to describe the flow of water over the land surface in two dimensions has been pivotal in enabling the development of large‐scale hydraulic models. A novel simplified implementation of the shallow water equations [*Bates et al*., 2010] yielded an algorithm for which the minimum stable time step scales linearly with decreasing grid size, rather than quadratically as had been the case with previous diffusion wave formulations [*Hunter et al*., 2005]. Furthermore, improvements to the software implementations of shallow water algorithms through parallelization on central and graphical processing units [*Kalyanapu et al*., 2011; *Lamb et al*., 2009; *Neal et al*., 2009, 2010; *Yu*, 2010] have yielded dramatic reductions in model runtimes. Together, these advances have provided a step change reduction in the computational resource required to undertake simulations, presenting an opportunity to simulate flooding on a global scale using the kinds of process‐rich hydraulic models normally used for more detailed localized studies.

One important limitation of standard 2‐D approaches over large domains is the inability to represent rivers whose width is considerably smaller than the grid size. For global models, where grid scales may be limited by terrain data resolution, subgrid methods and hybrid 1‐D/2‐D models have emerged as potential methods for representing such channels. *Neal et al*. [2012] tested four model structures for an 800 km reach of the River Niger in Mali: 1‐D only (no floodplain), 2‐D only (no channels), coupled 1‐D/2‐D (main channels with floodplain), and a 2‐D subgrid model (main channels, smaller subgrid floodplain channels, and floodplain). The study determined that inclusion of both the channel network and floodplain was essential, and that inclusion of the smaller subgrid channels on the floodplain yielded significantly increased simulation accuracy in terms of water level, wave propagation speed, and inundation extent.

An unavoidable complication of a global model is the range of topographies that will be encountered. The types of model used to simulate inundation are typically derived from the shallow water equations, themselves a simplification of the Navier‐Stokes equations made under the assumption of large horizontal length scale relative to vertical length scale, implying a small vertical velocity. This assumption is reasonable on most floodplains where the gradients are low, but in order to ensure model stability and conservation of mass it is necessary for the model to be able to handle the areas of steep or discontinuous terrain that will inevitably be encountered in a high‐resolution global model. Within channel, it is possible to represent inline nonshallow water features such as waterfalls and control structures using the empirical weir equation [*Ackerman et al*., 2008]. By using the weir equation to compute energy loss, model stability can be ensured in areas of steep flow but at the possible expense of solution accuracy in the water surface profile. However, this may be an acceptable compromise as flood hazard on steep terrain is likely to be limited and the critical issue is to conserve mass for conveyance to the floodplain. On the hillslope, a possible solution is to replace the shallow water equations with a simple routing scheme in areas where the topography is unsuitable for a shallow water type model. Such a scheme should convey water downslope in a mass‐conservative manner to ensure that the correct volume enters the floodplain, thus allowing the shallow water equations to take over and produce a realistic simulation of floodplain dynamics.

#### 5.2 Method

The hydraulic engine used here is based on the subgrid variant of LISFLOOD‐FP and employs the efficient inertial formulation of the shallow water equations [*Bates et al*., 2010; *Neal et al*., 2012]. The model is extended with a routing scheme [*Sampson et al*., 2013] that uses a slope‐dependent fixed velocity algorithm to calculate flow between adjacent floodplain cells in cases where the water surface slope is too steep to allow a stable solution to be calculated using the shallow water model. The routing scheme moves water according to a precalculated flow direction map based on elevation gradients between cells, and the velocity of flow is based on empirical data relating the velocity of overland flow to surface gradient produced by the United States Department of Agriculture [*Kent et al*., 2010]. Within channel, the weir equation is used in place of the shallow water equations in areas of steep terrain (a slope threshold of 5% is used to ensure stability).

Inundation dynamics on large rivers are simulated at 30 arc sec resolution rather than 3 arc sec resolution as DEM noise is reduced on the coarser grid, enabling a more stable estimate of water surface elevation to be produced over large floodplains. This has the additional advantage of reducing model runtimes by more than 2 orders of magnitude, allowing the long inundation events associated with seasonal floods on some major rivers to be simulated fully. A smooth water surface is then calculated by interpolating between points at the center of each 30 arc sec cell, enabling water heights to be reprojected onto the 3 arc sec DEM to obtain simulated water depths at the higher resolution. The decision to preserve water surface elevation, rather than mass, when downscaling was taken due to the large vertical noise error of the 3 arc sec SRTM DEM relative to the 30 arc sec SRTM DEM. A previous study has shown that downscaling flood extents onto a noisy DEM using a mass‐conservative method can lead to large local errors, and that under such conditions it may instead be preferable to preserve the water surface elevation simulated on the coarse, low noise, DEM while allowing some local extension to the downscaled flood extent where appropriate according to the high‐resolution topography [*Schumann et al*., 2014a]. For smaller channels and surface water inundation, where flows are driven by intense precipitation rather than the RFFA, simulations are undertaken directly on the 3 arc sec DEM. Results from the two types of simulation are merged to create the final hazard maps.

### 6 Automation Framework

#### 6.1 Method

The complexity and sheer size of a high‐resolution global flood model require that most elements of the model build process be automated. We developed a modular near‐automated model build framework augmented with python‐based GIS routines to generate suitable input data. The framework implements the methods outlined in the sections above and controls all stages of the hydraulic model build, execution, and postprocessing. A flowchart for the framework is provided in Figure 1. The two significant manual steps that remain are (1) building the databases of river widths using satellite imagery and (2) manual addition of major known defense structures. Being modular, the framework is easily updated as new data and methods become available to improve or supplement existing components.

## 2 Results

**Figure 3:**
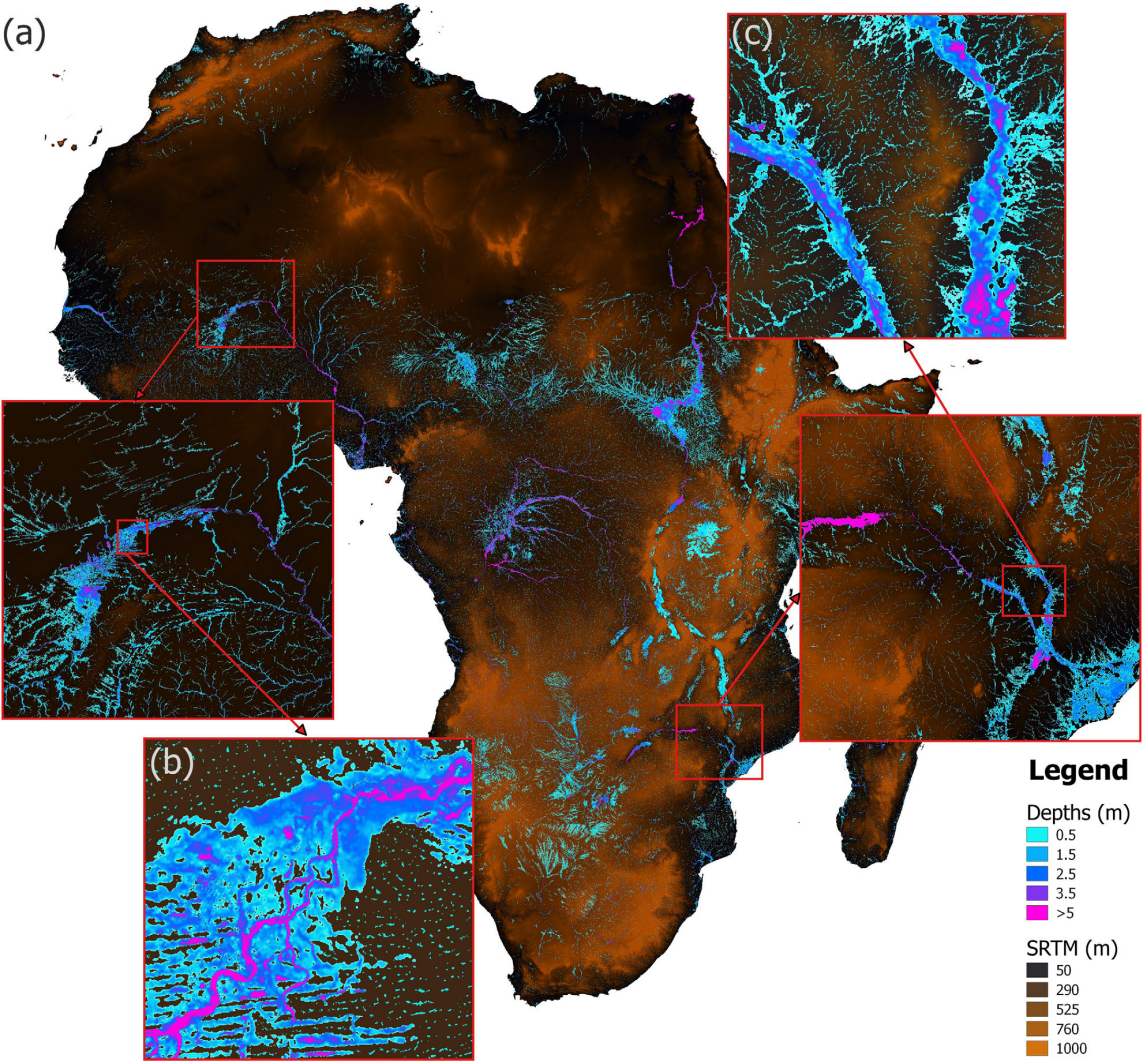
Example output from global flood model, showing 1 in 100 year maximum flood depth for (a) all of Africa, (b) Inland Niger Delta, and (c) Zambezi River floodplain.

The globally applicable model used here produces 3 arc second (∼90 m) resolution maximum water depth maps across the entire simulated global domain for 10 specified return period flows. The automation framework constructs tiles that are executed independently and then merged to form continuous data layers; the tiles have a 1° overlap across which a weighted blend is calculated to reduce the impact of anomalous boundary effects at the edge of each simulation domain. The total runtime for a typical 10° × 10° equates to approximately 2000 h on a single core of a 3.2 GHz Intel i7 processor, although the exact value for each tile varies according to several factors, including percentage land surface, climate (drier arid tiles are typically faster to simulate than wetter tropical tiles), and hydrograph durations on the largest rivers in the tile. As LISFLOOD‐FP is Open‐MP parallelized, and the reach decomposition approach enables multiple simulations to be run simultaneously, model runtime can be shortened significantly where sufficient computational power is available: it is typically possible to build, execute, and postprocess a single 10° × 10° tile in under 24 h using a 200 processor core cluster. An example of raw model output for a 1 in 100 year return period simulation across Africa is shown in Figure 3. A number of characteristic large‐scale hydrological features such as lakes, valleys, and coastal wetlands are immediately apparent upon initial visual inspection, with some being highlighted further in the figure. The existence of such features can be easily verified using freely available satellite imagery.

A more thorough validation of the performance of flood hazard maps is challenging as they do not attempt to recreate the characteristics of a single observable event, instead highlighting those areas at risk from plausible events of a given type and magnitude. A pragmatic approach to validating the results of a global model is to compare the results against an analogous regional data set constructed using high‐resolution models and detailed local data. This approach has precedence and was recently used to assess the performance of the aforementioned EC‐JRC model; we employ it here using local hazard maps from Canada and the United Kingdom as our benchmark data sets. Results from the United Kingdom are also compared directly with results from the EC‐JRC model. To the author's best knowledge, this validation is more substantive than any other yet published for a global flood hazard model, but it is still subject to some clear limitations in regard to the size and location of the test sites. Ideally, the model would be validated against a high‐resolution flood hazard layer from a large catchment in a data‐scarce region, but by definition such a data set does not exist as it would require a large amount of detailed data to produce. Our validation therefore focuses on catchments where data availability has allowed the creation of high‐quality benchmark flood hazard information (moderate‐sized, temperate, and continental catchments in the developed world), but it should be noted that no such local data (for example, enhanced terrain data or local flow records) were used to calibrate or otherwise modify the standard build procedure of the global model in the results presented below.

### 2.1 Performance Metrics

The performance measures used are spatial variants of the commonly employed Hit Rate, False Alarm Ratio, and Critical Success Index, the latter of which is often referred to within the hydraulic modeling community as the “Fit” statistic [*Alfieri et al*., 2013; *Aronica et al*., 2002; *Bates and De Roo*, 2000; *Fewtrell et al*., 2008; *Werner et al*., 2005]. A simple error bias metric is also used. The Hit Rate (*H*), sometimes referred to as the probability of detection, is a simple measure that indicates how well the model replicates the benchmark data without penalizing for overprediction:

(1) $$H=\;\frac{A_{m}\cap^{}A_{b}}{A_{b}}\;$$

where *A_m_* is the modeled inundated area and *A_b_* in the benchmark inundated area. *H* ranges from 0 to 1, with a score of 1 indicating that all wet cells in the benchmark data are wet in the model data. The False Alarm Ratio (*F*) is a measure of model overprediction (i.e., “false alarms”):

(2) $$F=\frac{A_{m}\backslash A_{b}}{\left(A_{m}\cap^{}A_{b}+A_{m}\backslash A_{b}\right)}$$

where scores range from 0 (no false alarms) to 1 (all false alarms). The Critical Success Index (*C*) extends on *H* and *F* to create a combined score that penalizes for both underprediction and overprediction:

(3) $$C\;=\;\frac{A_{m}\cap^{}A_{b}}{A_{m}\cup^{}A_{b}}\;$$

where scores range from 0 (no match between model and benchmark) to 1 (perfect match between benchmark and model). Finally, a simple measure of error bias (*B*) is used:

(4) $$B=\frac{A_{m}\backslash A_{b}}{A_{b}\backslash A_{m}}$$

where *B* varies between 0 and 1 indicates a tendency of the model to underpredict and a bias between 1 and ∞ indicates a tendency of the model to overpredict.

Results from the 3 arc‐second model runs were converted from their native raster format to a binary wet/dry shapefile format to allow the above metrics to be calculated. This was done in order to avoid resampling the higher resolution benchmark data to the lower model grid resolution.

In addition, aggregate performance metrics were also used. Many end users of global hazard models aggregate the data to a level that is commensurate with their operating scale. For example, metadata for insurance policies in the developing world are often poor with limited (or no) geocoding, resulting in considerable uncertainty about the physical location of an asset; commonly all that will be known is a high‐level postcode or administrative unit such as county or district. To give an indication of model performance under these conditions, the binary benchmark and simulated data for the catchment‐wide test cases are aggregated to a 30 arc sec (∼1 km) grid, with each pixel taking a value between 0 and 1 to represent the fraction of the cell flooded. The mean absolute error (*E_A_*) between aggregated and benchmark data is then calculated:

(5) $$E_{A}=\frac{\sum_{i=1}^{N}|M-B|}{N}$$

where *M* is modeled flooded fraction, *B* is benchmark flooded fraction, and *N* is number of cells. The error for each catchment is calculated under two conditions: (i) exclusive of cells that are dry in both benchmark and model and (ii) inclusive of cells that are dry in both benchmark and model. An aggregate error bias (*B_A_*) metric is also calculated:

(6) $$B_{A}=\frac{\sum_{i=1}^{N}M-B}{N}$$

where a positive error bias indicates a tendency of model overprediction relative to the benchmark data.

### 2.2 Benchmark Data Description

The Canadian benchmark data were supplied by the Informatics Branch of the Alberta State Government and consist of vector‐based binary wet/dry flood hazard maps for a selection of key urban centers in Alberta (Calgary, Edmonton, and Red Deer), each of which has a river running through it (the Bow, the North Saskatchewan, and the Red Deer River, respectively). These three rivers have their origins in the Rocky Mountains and flow through postglacial valleys on gravel beds. Flood hazard along these rivers is most severe in spring and summer when alpine snow melt can combine with spring rainfall or convectional summer storms to produce extreme discharge events. The data set was produced as part of the Alberta Flood Hazard Identification Programme by simulating 1 in 100 year design floods using steady state 1‐D Hydrologic Engineering Center River Analysis System (HEC‐RAS) models. The 1‐D water levels were reprojected onto the DEM using an AutoCAD/QuickSurf software package to create the 2‐D flood outline. The models were built using ground surveyed channel cross sections (including defenses) in conjunction with 1:5000 scale 1 m contour maps; design flows were derived from federal flow records from the Water Survey of Canada (part of Environment Canada). The Alberta data set has limited coverage as HEC‐RAS models were only built for certain river reaches and we therefore limit our analysis to the corresponding domain areas of the benchmark data. It was also necessary to mask a number of minor channels present in the global model but not in the benchmark data to ensure a fair comparison. This was generally straightforward as only major tributaries were modeled within the benchmark data; where minor tributaries were clearly missing from the benchmark data the corresponding channels were removed from the global model output.

The data from the United Kingdom comprise catchment‐wide 1 in 100 year flood outlines for the Severn and Thames catchments (∼11,000 and ∼16,000 km^2^, respectively) and were supplied by the Environment Agency of England and Wales as shapefiles. The data are multisource and are composed, in descending order of preference, of (a) flood extent observations of events with an estimated return period of ∼100 years; (b) detailed 1‐D or 1‐D/2‐D hydraulic models build using airborne LIDAR data with a spatial resolution of ∼2 m and a vertical accuracy of <10 cm; (c) a 2‐D hydraulic model constructed using airborne interferometric synthetic aperture radar (IfSAR) terrain data with a spatial resolution of ∼5 m and vertical accuracy of ∼0.5–1 m. Flows for the hydraulic models were derived using flood frequency analysis methods based on extensive flow records [*Institute of Hydrology*, 1999] and defenses were generally assumed to have failed (although in the case of observed flood outlines, this assumption may not be valid). These catchment‐wide flood outlines provide extensive coverage and thus minimal masking of the global data was required as most channels simulated using the global method were also present in the benchmark data. Last, in order to allow a direct comparison with the aforementioned pan‐European EC‐JRC model, a second mask was created that removed all channels with upstream areas of less than 500 km^2^ as this constraint was imposed by the EC‐JRC model [*Alfieri et al*., 2013]. This same mask was applied to the raw model output from the earlier study to ensure a consistent comparison.

The benchmark data sets employed in this study have been selected as they are representative of the types of flood hazard information currently available to end users in some more developed regions. However, the local engineering‐grade models used to create the benchmark data are not themselves error free, being subject to data and methodological limitations, and correlations between the benchmark and global model errors are unknown. While such data remain the best available way to assess the performance of the global hazard model, the analyses that follow should be viewed in the context of these limitations.

### 2.3 Canada

**Figure 4:**
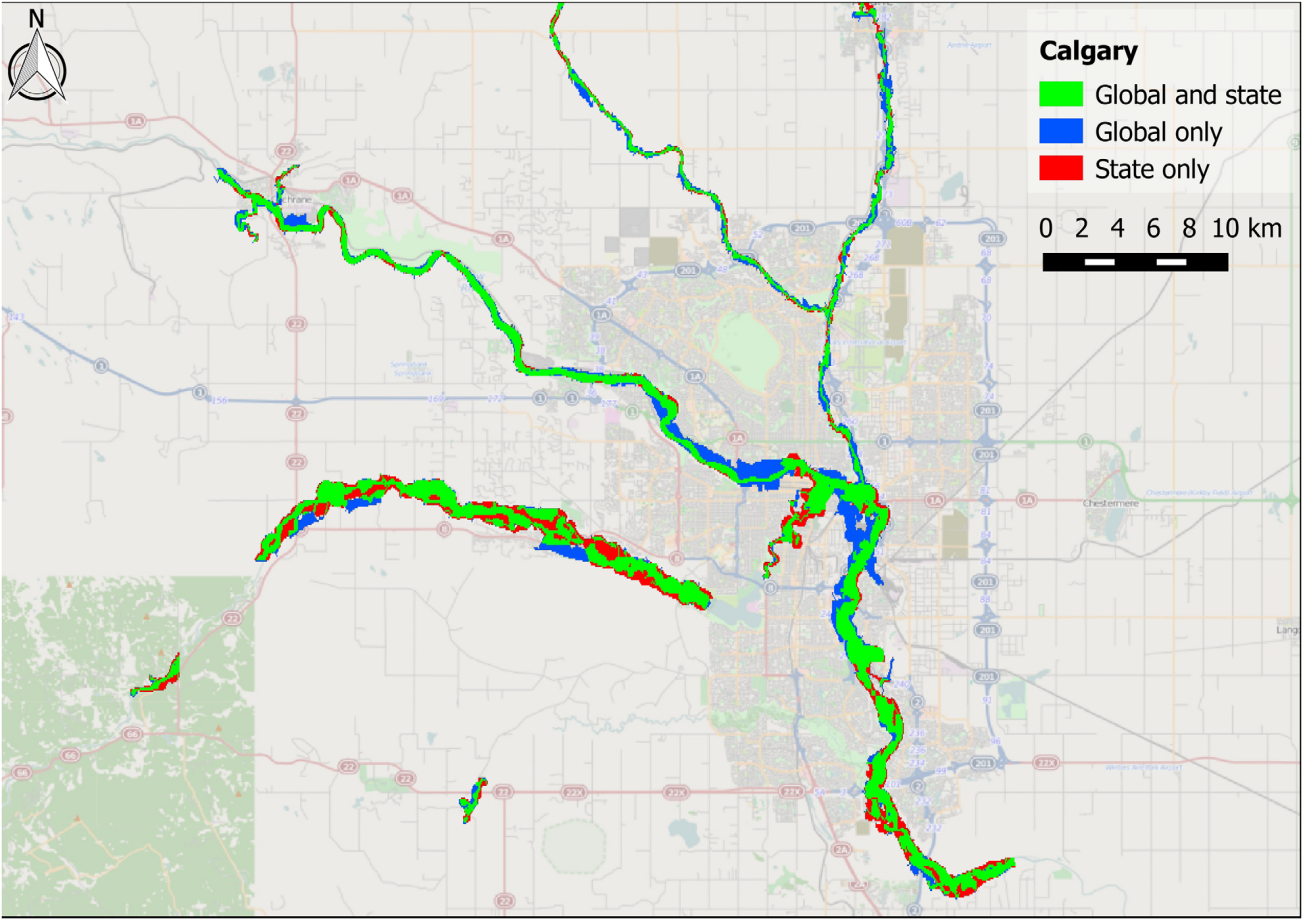
Map showing fit between global model and Canadian state benchmark data for the Bow River (and its main tributaries) in Calgary, Alberta. Green shading represents matching flooded area in both benchmark data and the global model; blue shading represents flooded area unique to the global model; and red shading represents flooded area unique to the benchmark data.

**Figure 5:**
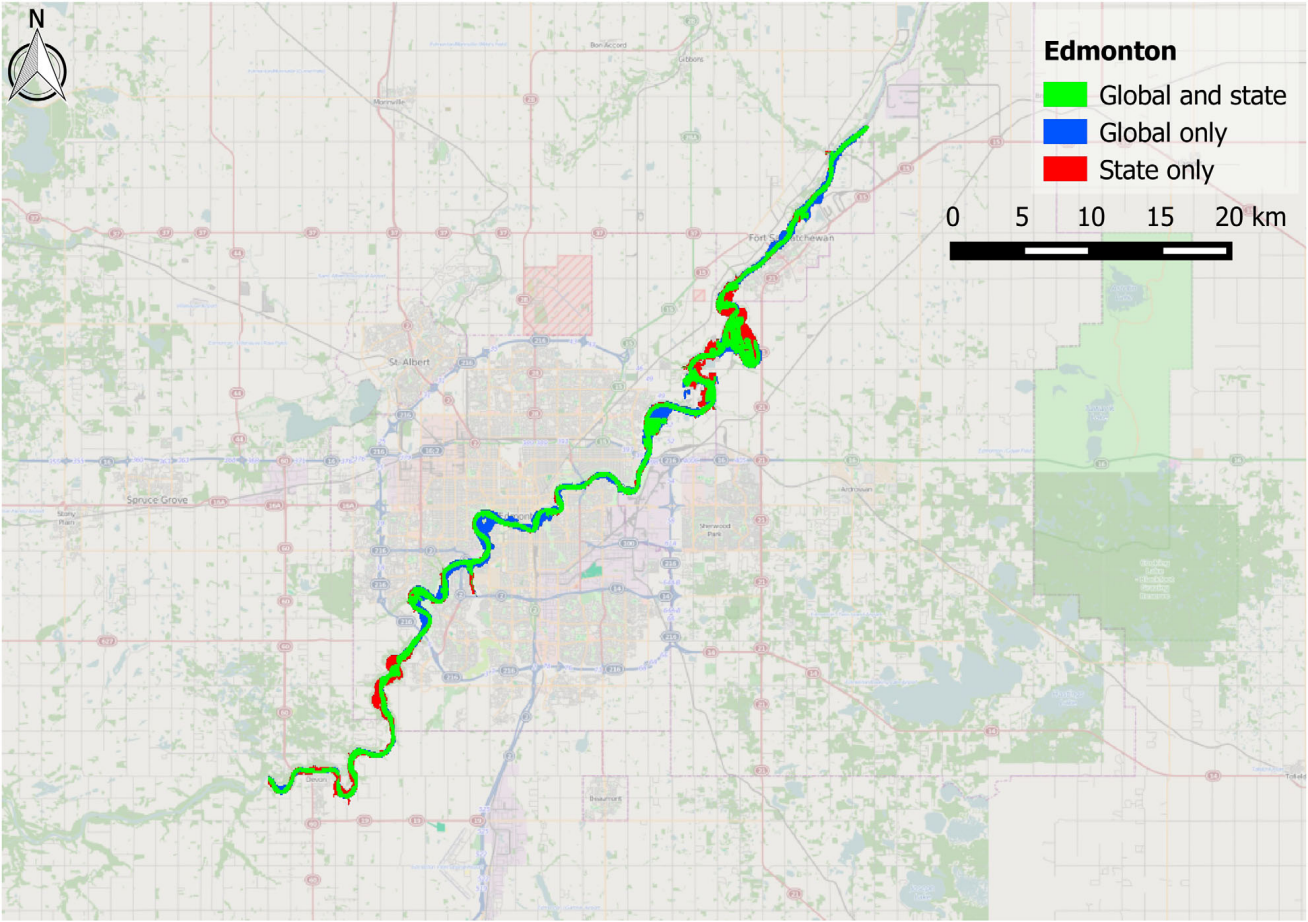
Map showing fit between global model and Canadian state benchmark data for the North Saskatchewan River (and its main tributaries) in Edmonton, Alberta. Green shading represents matching flooded area in both benchmark data and the global model; blue shading represents flooded area unique to the global model; and red shading represents flooded area unique to the benchmark data.

**Figure 6:**
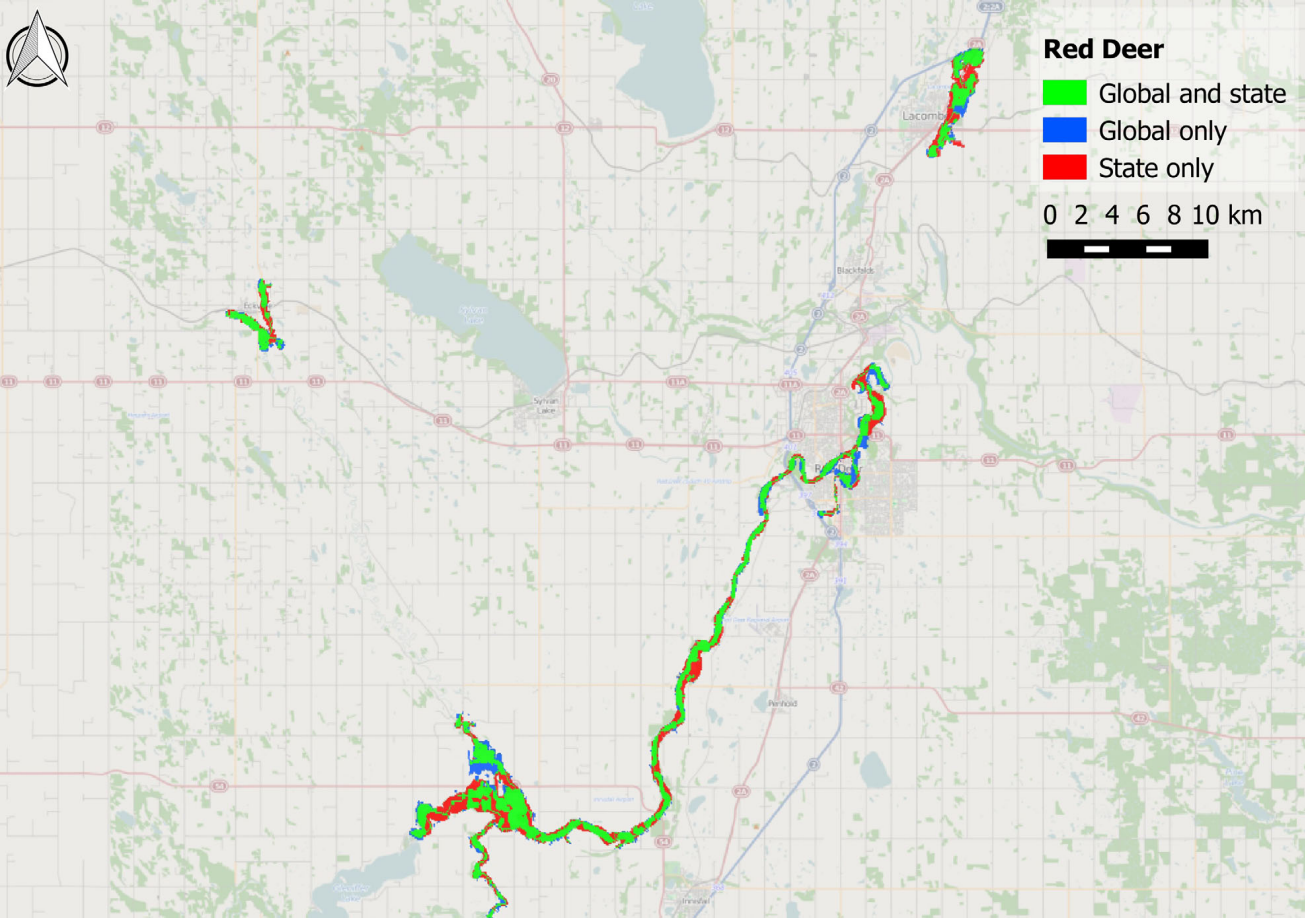
Map showing fit between global model and Canadian state benchmark data for the Red Deer River in Red Deer, Alberta. Green shading represents matching flooded area in both benchmark data and the global model; blue shading represents flooded area unique to the global model; and red shading represents flooded area unique to the benchmark data.

The Alberta State flood hazard maps are centered around three settlements: Calgary, Edmonton, and Red Deer. These test cases pose a stern challenge for a global model as they represent moderate size rivers flowing through urban areas. The performance metrics for these three areas are shown in Table 2, along with the upstream area of the largest channel in each test case to provide an indication of river size. The simulated reach length and number of surveyed cross sections used to produce the benchmark data are also shown. Comparisons between benchmark and modeled data are shown visually in Figures 4, 5, 6.

**Table 2:** Model Performance Metrics for the 1 in 100 Year Test Case Reaches in the State of Alberta, Canada

Model	Benchmark Reach Length (km)	Benchmark Cross Sections	Max Upstream Area (km^2^)	Hit Rate	False Alarm Ratio	Critical Success Index	Error Bias
Calgary (Bow River)	∼78	145	∼15,000	0.75	0.26	0.6	1.07
Edmonton (North Saskatchewan River)	∼93	115	∼17,000	0.81	0.22	0.65	1.2
Red Deer (Red Deer River)	∼60	55	∼44,000	0.67	0.18	0.59	0.45

The *H* score of the global model on the benchmark data varies between 0.67 and 0.81, indicating that the global model is able to capture at least two thirds of the area designated as being at risk by the Alberta State 1 in 100 year flood hazard map. When additionally penalizing for overprediction using the *C* score, the performance decreases to between 0.59 and 0.65. Figures 4, 5, 6 provide a visual reference of model performance, and the broad agreement between benchmark and global flood hazard maps is apparent. Both Calgary and Edmonton show larger differences between *H* and *C* than Red Deer, and the tendency of the global model to overpredict relative to the benchmark data is particularly evident in the blue areas visible around the center of Calgary in Figure 4. This behavior may be explained by the global model's reduced ability to resolve objects on the floodplain that restrict flow, such as high‐density urban developments in city centers, but some of the additional flooding seen in central Calgary may also represent a limitation of the one‐dimensional hydraulic model used to produce the benchmark data. Such a model would have been limited by the width of the cross sections used to construct the model domain, and it is therefore not possible to ascertain the cause of this discrepancy between the global and benchmark data. The relatively small amount of underprediction that occurs within urban areas across the three test cases indicates that the filter employed to reduce the positive elevation bias typically present in SRTM data is functioning well; previous studies that simulate flood hazard in urban areas using SRTM have found severe underprediction of flooded area to occur as a result of the data representing roof top rather than ground elevations [*Alfieri et al*., 2013; *Sanders*, 2007]. The Red Deer model is the most rural of the three Canadian test cases and is the only one whose errors are biased toward underprediction. This occurs because the majority of flooded area within the benchmark data corresponds to extensive braid bars in the wide main channel. These braid bars are forested, generating a positive elevation bias in the SRTM data that is not removed by the SRTM postprocessing algorithm employed by the global model. This creates a falsely elevated model floodplain and corresponding reduction in flood extent.

### 2.4 UK

**Figure 7:**
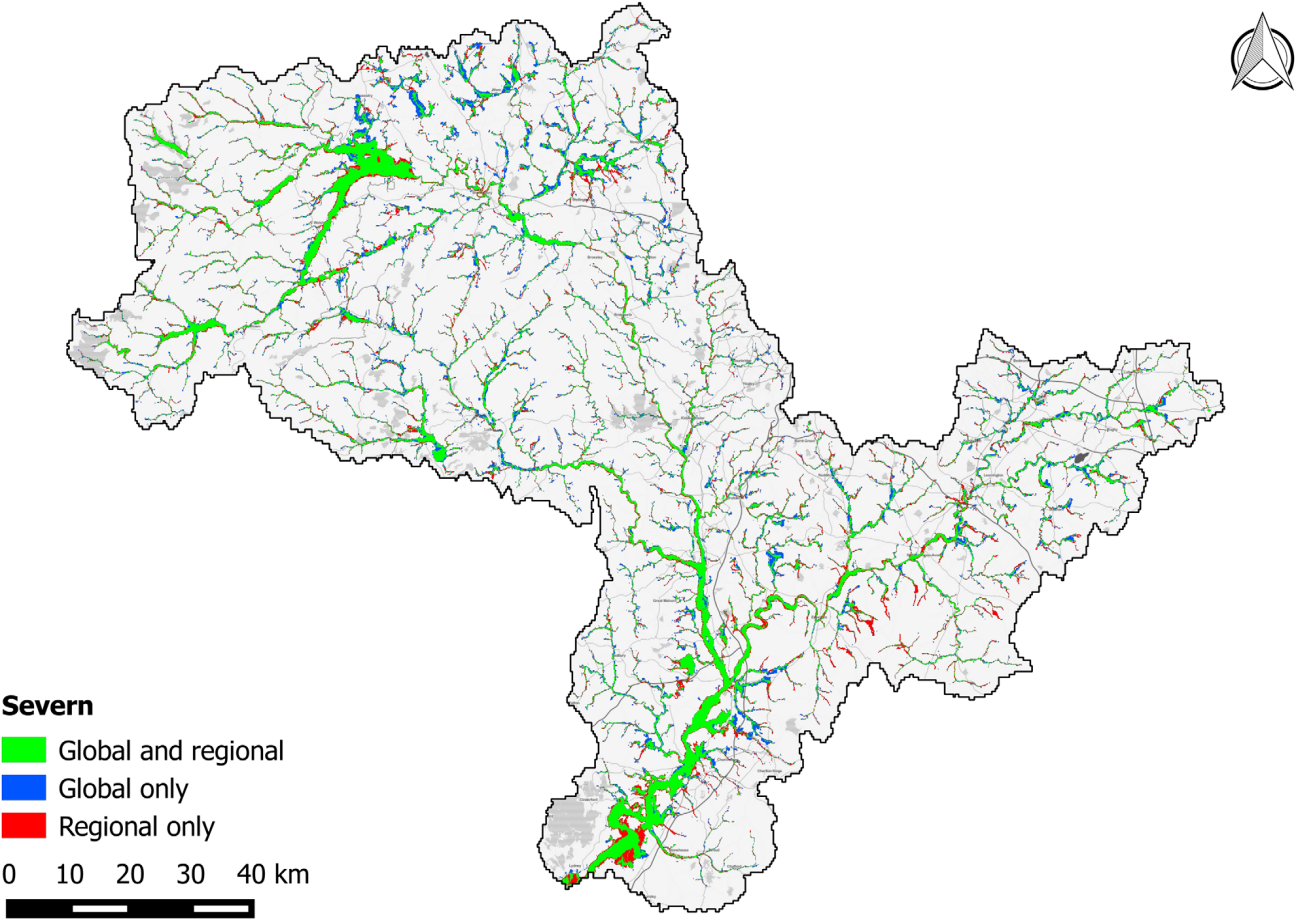
Map showing fit between global model and UK Environment Agency benchmark data for the Severn catchment (∼11,000 km^2^). Green shading represents matching flooded area in both benchmark data and the global model; blue shading represents flooded area unique to the global model; and red shading represents flooded area unique to the benchmark data.

**Figure 8:**
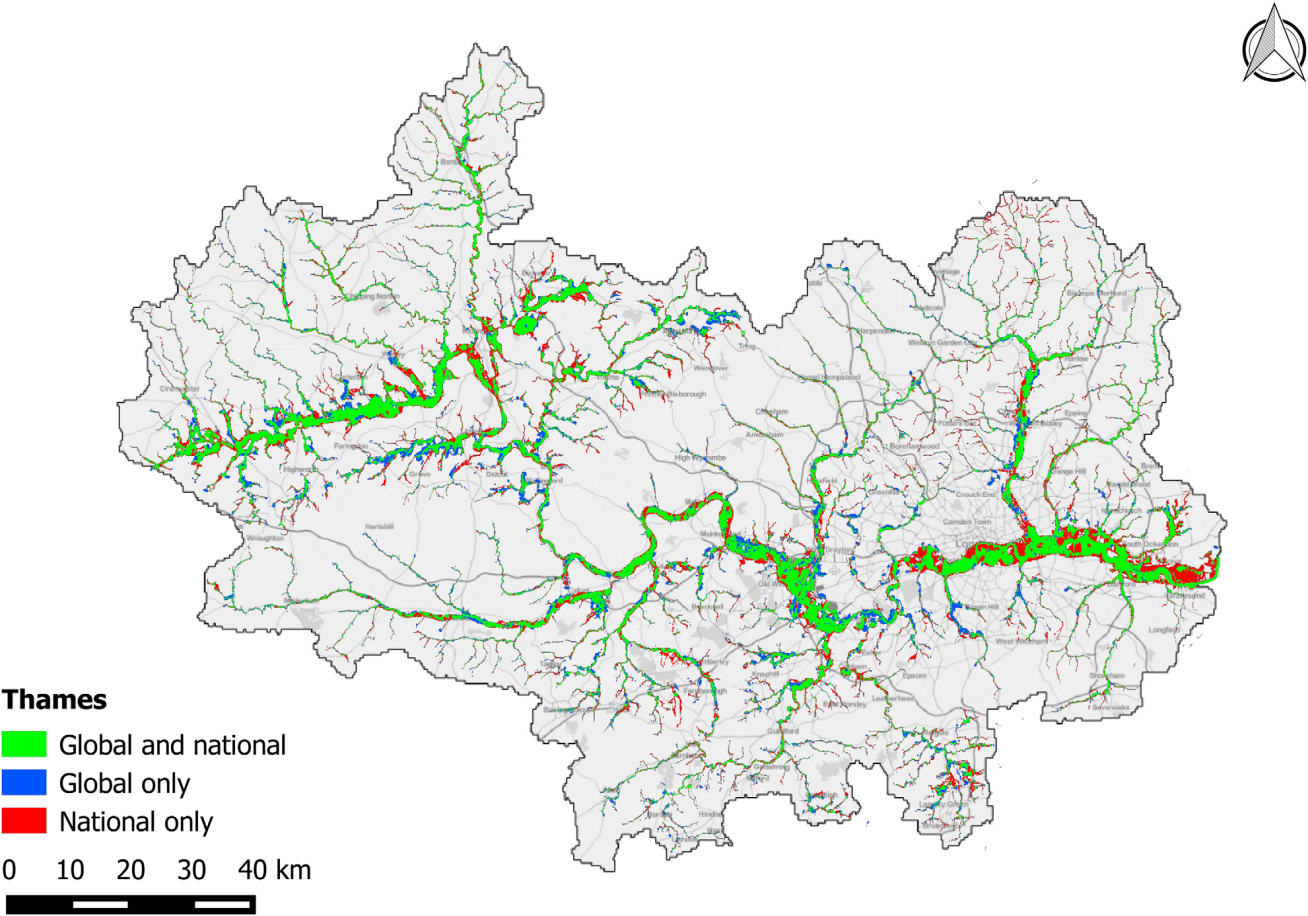
Map showing fit between global model and UK Environment Agency benchmark data for the Thames catchment (∼16,000 km^2^). Green shading represents matching flooded area in both benchmark data and the global model; blue shading represents flooded area unique to the global model; and red shading represents flooded area unique to the benchmark data. The present omission of tidal flooding within the global model is visible in the underprediction of flood hazard relative to the benchmark data in the lower reach of the Thames.

The performance metrics for the Thames and Severn catchments are presented in Table 3, with visual comparisons between benchmark and global data sets shown in Figures 7 and 8. In addition to the standard model build, performance metrics are shown for two additional model variants. The first of these are 2‐D only models in which the subgrid channel structures have been removed and the inflow hydrographs reduced by a commensurate estimate of bankfull discharge that is then applied directly to the floodplain. The second additional set of models exclude flooding from channels with an upstream area of less than 500 km^2^ in order to allow performance on larger channels to be considered separately, and to enable a direct comparison with the EC‐JRC model. Performance metrics for the EC‐JRC model have been recalculated using this study's methods and are also shown in the table; small discrepancies between the values presented here and in the original publication are due to (i) slight differences in the 500 km^2^ mask and (ii) regridding of the benchmark data onto a 90 m raster grid in the earlier study.

**Table 3:** Model Performance Metrics for the Severn and Thames Catchments in the UK Relative to the Benchmark UK Environment Agency Hazard Data

Model	3 arc sec Resolution				30 arc sec Resolution		
	Hit Rate	False Alarm Ratio	Critical Success Index	Error Bias	Aggregate Error (Wet Cells)	Aggregate Error (All Cells)	Aggregate Error Bias
Thames (standard model build)	0.65	0.45	0.43	1.47	0.05	0.04	0.01
Severn (standard model build)	0.74	0.49	0.43	2.61	0.05	0.04	0.03
Thames (2‐D only)	0.81	0.58	0.38	5.82	0.10	0.08	0.08
Severn (2‐D only)	0.85	0.62	0.36	10.92	0.10	0.08	0.09
Thames (>500 km^2^)	0.73	0.3	0.56	1.14	0.09	0.02	0.01
Severn (>500 km^2^)	0.83	0.23	0.67	1.45	0.05	0.01	0.01
Thames (2‐D only, >500 km^2^)	0.83	0.39	0.54	3.07	0.11	0.02	0.07
Severn (2‐D only, >500 km^2^)	0.87	0.36	0.58	3.4	0.08	0.02	0.06
Thames (JRC^a^; >500 km^2^)	0.66	0.42	0.45	1.43	0.16	0.03	0.03
Severn (JRC^a^; >500 km^2^)	0.75	0.33	0.55	1.45	0.1	0.02	0.02

a See *Alfieri et al*. [2013] for model details.

It is apparent from Table 3 that model performance is consistently better on the Severn than on the Thames, and that model performance declines significantly when smaller channels are considered relative to when only larger channels are considered. The difference in skill between the Thames and Severn has several explanations. The Severn is a relatively simple catchment in terms of topography, with the majority of its larger floodplains consisting of rural pasture. In comparison, the Thames catchment poses a stern modeling challenge as it features complex chalk hydrology and is heavily urbanized, flowing through a number of sprawling towns upstream of London before continuing through the center of the capital itself. As a result, the SRTM terrain model is more closely matched to the high‐quality terrain data used for the benchmark hazard maps over the Severn catchment than it is over the Thames. Furthermore, flooding along the Thames is prevented or reduced by a number of substantial flood defense and alleviation schemes that are more extensive than any modifications made to the Severn and its tributaries. Visual inspection of Figure 8 shows the global model to underestimate flood hazard in the eastern part of the Thames catchment. This is due to an assumed sea level of 0 m (i.e., mean sea level) in the global model whereas the Environment Agency flood hazard map assumes a 1 in 200 year coastal flood along with failure of the Thames tidal barrier, thus exposing a large area of central London to tidal surge flooding.

Both models exhibit relatively good skill on larger channels, with *C* scores of 0.56 and 0.67 for the Thames and Severn, respectively. To put these score into context, previous (50 m) spatial resolution event‐based modeling studies of smaller rural reaches of the Thames and Severn achieved maximum *C* scores against remotely sensed observed extents of 0.72 and 0.65, respectively [*Aronica et al*., 2002; *Horritt and Bates*, 2002]. A subsequent model of a 16 km rural reach of the Severn, built using LIDAR terrain data resampled to 18 m resolution and calibrated using 1.2 m resolution airborne SAR, achieved a *C* against SAR‐derived observed extents of between 0.72 and 0.89 depending on the date of comparison [*Bates et al*., 2006]. Given that these models employed higher‐quality topographic data (photogrammetry and LIDAR), gauged flow data, surveyed bathymetry and calibration to optimize friction parameters for local conditions, the relative skill of the global model is encouraging. The reduction in model performance over smaller catchments is unsurprising given the ∼90 m resolution and poor vertical accuracy of the SRTM data used in global study. The benchmark data are constructed using DEMs of (at worst) 5 m resolution with ∼1 m vertical accuracy, enabling them to capture small‐scale topographic features that are smaller than the individual pixels of the global model. The result of this is that topography appears smoothed in the global model, often leading to an overprediction of flood extents as demonstrated in previous studies [*Fewtrell et al*., 2008; *Sampson et al*., 2012; *Yu and Lane*, 2011]. Other errors relating to the limited vertical precision of the SRTM data are also more pronounced for smaller channels, such as channel location errors. Noise in the DEM may cause small channels to be located incorrectly, as can pixilation effects owing to the limited resolution; this can adversely affect model performance statistics when compared to a higher resolution benchmark data set. However, despite the significant fall in *C*, *H* remains quite high indicating that the model is indicating flood hazard in broadly the correct areas and that model output may still be valuable for applications where a higher *F* is acceptable.

Comparison of the full complexity global model relative to the simpler 2‐D only global model demonstrates a critical limitation of the simpler approach. The 2‐D only models consistently produces a higher *H*, but this is because the models are severely overestimating flood extents as demonstrated by lower *C* scores, high *F* scores, and large positive error biases. This overprediction occurs because (i) noise or lack of resolution can cause flow blockages in the DEM that result in elevated water levels and increased flood extent and (ii) water velocities are low due to the lack of deep channels, again resulting in elevated water levels. Both of these problems are addressed by the subgrid scheme in which faster in‐channel flow is simulated and local topographic errors can be readily overcome by forcing monotonically decreasing channel bed elevations.

Of the three model variants that only consider channels with an upstream area of >500 km^2^, the subgrid‐enabled global model provides the closest match to the benchmark data. The other two models are both 2‐D only and yield similar *C* scores on the Severn catchment, with the EC‐JRC model having a lower overprediction bias than the simplified 2‐D only global model; these differences are likely to be due to the different methods used by the two models to generate hydrographs. However, on the Thames, the EC‐JRC model is significantly outperformed by both variants of the global model. As noted in the original analysis of the EC‐JRC model, the challenge of using a hydrological model to derive extreme event hydrographs for the complex chalk‐based Thames catchment may offer a partial explanation. However, some of the underprediction exhibited by the EC‐JRC model within London is also due to the urban elevation bias of SRTM, something that is alleviated by the simple SRTM urban filter used by the global model presented here.

Model performance when aggregated to a ∼1 km grid is strong, and the small aggregate errors show a similar pattern between the model structures, with the full complexity global model outperforming the 2‐D only variants that again show a tendency toward overprediction. However, the difference between small and large catchments is not evident; this is because for minor channels, the area of flooding relative to the total pixel area is small, leading to a small aggregate errors. Furthermore, where the pixel‐by‐pixel comparison penalizes imperfect alignment in flooded areas caused by errors in the SRTM terrain data, the aggregate comparison only penalizes if the offset is large (i.e., if there is a bias).

## 3 Discussion

This paper presents a schematic framework for constructing a high‐resolution (3 arc sec or ∼90 m) global flood model by leveraging a number of recent advances in remote sensing and hydrology. A key constraint when developing the framework was to ensure that all data sets required for its construction had near‐global coverage as many of those traditionally required to build and calibrate flood hazard models (for example rainfall and river discharge records) are relatively scarce outside western Europe and North America (e.g., GRDC) [*Chen et al*., 2002]. The approach uses regionalized flood frequency analysis in place of hydrological models in order to generate return period design flood hydrographs across the global river network. A two‐dimensional hydraulic model is used to propagate the hydrographs across an SRTM‐derived DEM that has been processed to reduce elevation biases caused by vegetation and buildings. Although not implemented in this study, the model can also account for the effects of flood defenses by modifying the channel conveyance in urban areas to allow an estimated return period discharge to be contained. The defense method involves using a simple regression‐based model that relates satellite luminosity and GDP to defense standard to estimate an appropriate return period conveyance for a given site; it will be more fully explored in a future study.

The setup presented here differs from previously published global flood models in that it attempts to model inundation dynamics using a two‐dimensional hydraulic model operating at resolutions commensurate with the highest quality existing global terrain data sets. Its closest existing published counterpart is the pan‐European EC‐JRC model, but two key differences exist between the two approaches. The first of these is that the method presented here challenges the assumption that a hydrological model is required to drive a large‐scale hydraulic model due to the lack of suitable data for calibration and validation globally, instead opting to apply a regionalized global flood frequency analysis. Although a flood frequency approach has been used, this study does not make any comparison between the methods used to derive discharge for global scale models; such a comparison is beyond the scope of this study. Work comparing the performance of these methods across data‐rich and data poor regions represents an area of research that needs to be undertaken in the near future. However, the application of a flood frequency analysis presents an advantage in that estimates of discharge can be easily coupled to channel size.

This links to the second key difference which is the explicit inclusion of a detailed river channel network, parameterization of which is tied to the flood frequency analysis to ensure that discharge estimates are commensurate with channel geometry estimates at any given point on the network. This is unique as previous studies at continental or global scales have either used 1‐D routing schemes with a subsequent postprocessing approach to derive flood depths or extents [*Winsemius et al*., 2013; *Yamazaki et al*., 2011], or they have simulated flood wave propagation across the floodplain having first removed a volume of water corresponding to a channel conveyance estimate [*Alfieri et al*., 2013]. The explicit inclusion of channels has been shown to be crucial to the ability of a model to simulate large‐scale floodplain dynamics [*Neal et al*., 2012], and the higher performance of the global model relative to both a simplified 2‐D‐only version of itself the EC‐JRC model supports this finding.

The model framework described in this paper is presented as a “first generation” global flood hazard model and as such is subject to a number of uncertainties and limitations that should be addressed by future work. In our opinion, the dominant data uncertainty in such a model remains the quality of terrain data available for use. The shortcomings of the SRTM data set are well documented [e.g., *Reuter et al*., 2007; *Rexer and Hirt*, 2014], and despite a large body of work delivering significant improvements to the data since its original release over a decade ago, it remains poor relative to the higher‐quality LIDAR and airborne IfSAR data sets that are available over more limited areas and which are typically used for flood modeling [*Schumann et al*., 2014b]. Large and poorly characterized uncertainties are also present in the estimation of channel widths and depths across the global river network; at present, these are inferred from estimated bankfull river discharges but the ongoing development of global river width databases and future research efforts to estimate river channel depths from space borne observation of water levels [*Durand et al*., 2010] may alleviate these uncertainties over time. Such assimilation methods may also allow spatially variable channel friction parameters to be estimated remotely. Coupled to this are uncertainties in the estimates of bankfull and return period discharges produced by the global regionalized flood frequency analysis, and future work should seek to quantify the effect of these uncertainties on predicted flood extents. The method used to produce estimates of extreme rainfall intensities required for the simulation of flash flood hazard in small catchments is similarly uncertain and will miss the influence of local features in some cases. Such methods, along with their counterpart global hydrological models, should improve over time if discharge and precipitation records increase in length and number, although this will require developing nations to invest heavily in their hydrological monitoring capabilities. Of concern to many parties interested in the practical application of such models (e.g., the insurance industry) will be the significant uncertainties in flood defense representation. At present, a database of global flood defenses does not exist, and a concerted effort to begin putting one together would yield valuable data with which basic approaches that attempt to produce spatial estimates of flood defense standards globally, such as the one suggested in this paper, could be rapidly improved. The flood alleviation impacts of lakes and reservoirs would need to be included within this work as these are currently omitted from the model. One structural limitation of the model presented here that could be addressed at present is the lack of a tidal flood hazard component. Models that describe coastal surge heights exist and such tools have been used to drive hydraulic flood models before [*Hinkel et al*., 2014; *Lewis et al*., 2013]. The framework itself is structured to allow the future addition of a coastal component, and such an addition would likely reduce the underestimation of flood hazard in coastal areas that is currently exhibited by the model.

By considering both the performance of the model and the known limitations outlined above, it is possible to comment on the applicability of the model in its present form. The model has been demonstrated to perform well for inland flooding on smaller rivers than those previously associated with global flood hazard models (for example, the GLOFRIS model is limited to Strahler order 6 or greater), and the terrain correction applied to SRTM also enables the model to simulate flood hazard on vegetated and urbanized floodplains. However, the model is likely to underestimate flood hazard in coastal areas that are prone to tidal flooding due to the lack of a storm surge component. The model may also overestimate hazard along rivers with significant reservoir management capacity as such features are not yet incorporated. Finally, the effect on the model of extremely high infiltration rates in some arid and semiarid areas is as yet unknown due to a lack of suitable validation data, and therefore model results from these regions should be interpreted with care.

## 4 Conclusions

Validation of a flood hazard map is challenging as such data do not attempt to describe any single real event but instead attempt to describe the areas affected by all events of a certain magnitude. Previous global flood models have operated at resolutions that are too coarse to allow meaningful comparison to high‐quality local or national data, but the model presented here is sufficiently resolved to enable this approach. We present quantitative metrics that describe the global model's ability to replicate the flood extents shown by benchmark data sets from Canada and the UK. These metrics indicate that the global model is typically able to capture between two thirds and three quarters of flooded area in the local benchmark data, and that along nontidal reaches of large rivers, the skill is likely close to that of local models. Perhaps surprisingly, the model produced similar performance metrics in Canadian urban test cases as a result of the preprocessing of the underlying SRTM terrain data to reduce the elevation biases induced by radar returns from the tops of buildings. Skill relative to the benchmark data sets is observed to decrease along smaller channels as more subtle topographical features are unresolved in SRTM data and noise in the DEM can often lead to errors in the location of streams and minor tributary rivers within the model. The general smoothing of small topographical features also offers an explanation for the tendency of model errors to be biased toward overprediction.

Many practical applications of global hazard data sets such as this involve working at relatively coarse scales in conjunction with other global data sets that may have considerable spatial uncertainties. As it is common practice to aggregate hazard data to a scale that is commensurate with the operating scale of the application, the performance of the model when aggregated to a ∼1 km grid was also calculated for the UK whole‐catchment test cases. Under these conditions, model performance was strong, with mean absolute error in flooded fraction typically below 5% across both the Thames and Severn relative to similarly aggregated benchmark data. The aggregate performance indicates potential for this type of model to be used for preliminary flood hazard assessment in areas where more detailed local data are not available. However, at smaller scales, topography remains the single largest limiting factor in models of this type, and the forthcoming release of new higher resolution terrain sets, such as global public release of 1 arc sec SRTM and the TanDEM‐X Elevation12 DEM, offer exciting prospects for improvement in the near future.

**Erratum**

In the originally published version of this article, author Paul D. Bates's middle initial was incorrect. This has since been corrected and this version may be considered the authoritative version of record.
